# Understanding non-performance reports for instrumental activity of daily living items in population analyses: a cross sectional study

**DOI:** 10.1186/s12877-016-0235-0

**Published:** 2016-03-08

**Authors:** Margaret G. Stineman, Dawei Xie, Qiang Pan, Jibby E. Kurichi, Debra Saliba, Sophia Miryam Schüssler-Fiorenza Rose, Joel E. Streim

**Affiliations:** Department of Physical Medicine and Rehabilitation, Perelman School of Medicine, University of Pennsylvania, Philadelphia, PA 19104 USA; Department of Biostatistics and Epidemiology, The Center for Clinical Epidemiology and Biostatistics, Perelman School of Medicine, University of Pennsylvani, 423 Guardian Drive, 617 Blockley Hall, Philadelphia, PA 19104 USA; Anna and Harry Borun Chair in Geriatrics and Gerontology at UCLA, RAND, Santa Monica, CA USA; Research Physician, VA GLAHS GRECC, RAND, Santa Monica, CA USA; UCLA/JH Borun Center for Gerontological Research, RAND, Santa Monica, CA USA; RAND Health, RAND, Santa Monica, CA USA; Spinal Cord Injury Service, Veterans Affairs Palo Health Care System, 3801 Miranda Ave, Palo Alto, CA 94304 USA; Department of Neurosurgery, Stanford University School of Medicine, Stanford, CA USA; Geriatric Psychiatry Section of the Department of Psychiatry, Perelman School of Medicine, University of Pennsylvania, Philadelphia, PA USA; VISN 4 Mental Illness Research Education & Clinical Center, Corporal Michael J. Crescenz Veterans Affairs Medical Center, Philadelphia, PA USA

**Keywords:** Disparities, Function, Self-rated health, Survey design

## Abstract

**Background:**

Concerns about using Instrumental Activities of Daily Living (IADLs) in national surveys come up frequently in geriatric and rehabilitation medicine due to high rates of non-performance for reasons other than health. We aim to evaluate the effect of different strategies of classifying “does not do” responses to IADL questions when estimating prevalence of IADL limitations in a national survey.

**Methods:**

Cross-sectional analysis of a nationally representative sample of 13,879 non-institutionalized adult Medicare beneficiaries included in the 2010 Medicare Current Beneficiary Survey (MCBS). Sample persons or proxies were asked about difficulties performing six IADLs. Tested strategies to classify non-performance of IADL(s) for reasons other than health were to 1) derive through multiple imputation, 2) exclude (for incomplete data), 3) classify as “no difficulty,” or 4) classify as “difficulty.” IADL stage prevalence estimates were compared across these four strategies.

**Results:**

In the sample, 1853 sample persons (12.4 % weighted) did not do one or more IADLs for reasons other than physical problems or health. Yet, IADL stage prevalence estimates differed little across the four alternative strategies. Classification as “no difficulty” led to slightly lower, while classification as “difficulty” raised the estimated population prevalence of disability.

**Conclusions:**

These analyses encourage clinicians, researchers, and policy end-users of IADL survey data to be cognizant of possible small differences that can result from alternative ways of handling unrated IADL information. At the population-level, the resulting differences appear trivial when applying MCBS data, providing reassurance that IADL items can be used to estimate the prevalence of activity limitation despite high rates of non-performance.

## Background

The ability to perform instrumental activities of daily living (IADLs) predicts important health outcomes and is frequently assessed in geriatric practice, research, and surveys of population health. However, when compared to basic activities of daily living (ADLs), a larger number of respondents typically report non-performance of IADLs for non-health reasons. Little is known about the best approach to coding this response when IADL data are used to estimate population abilities. IADLs are important in interdisciplinary geriatrics, rehabilitation, healthcare, and policy applications because they measure the tasks people must be able to perform or have performed for them if they are to live safely in the community [1].

Recognizing the growing importance of functional assessment in policy and clinical practice, five stages of IADLs were established to group people according to increasing difficulty performing these tasks [2]. The stages express an underlying continuum of human functioning [1] and reflect the degree to which people experience difficulties in each of 6 activities. Compared to traditional counts of limitation, IADL stages define what activities people are still able to do without difficulty based on the International Classification of Functioning, Disability and Health participation in social roles and community activities [3]. IADL stages have been shown to be strongly related to age, perceptions of unmet needs for accessibility features in the home, and the presence of certain disabling conditions. IADL stages were also shown to be strongly predictive of one-, five-, and 10-year mortality and perceptions of reduced care quality [4–7]. Those at intermediate IADL stages were most likely to have a history of multiple falls [8].

The stage definitions [2, 6] range from IADL-0 (least) to –IV (most limited), with stage III containing people who do not fit the most typical hierarchy of abilities.

Many population surveys, including the Medicare Current Beneficiary Survey (MCBS), allow respondents to opt out of rating IADLs by first responding that they do not do them and then answering whether this was because of a health or physical problem or because of a different reason. If the respondent reports non-performance for a health reason, they are classified as having difficulty [9]. If, however, the respondent reports that non-performance is for another reason, effectively opting out of the question, it is not clear how to classify the response. It is possible that the non-performance represents a preference (to rely on performance by others) or lack of experience in an otherwise able individual. On the other hand, how do we know when people say their non-performance is not related to a health problem that there is not really an underlying health reason? It is possible that the individual has health or functional limitations that would prevent the completion of the activity even if attempted. If end-user clinicians, health service researchers, epidemiologists, or policy analysts are to use IADL stages to describe the status of their populations, it is necessary to choose a strategy or convention to handle this unrated or missing information. The assignment and implications of those who opt out of doing an IADL for other reasons is unclear. Those who do not do an IADL for reasons other than health or physical problems could be excluded from analyses, placed in the “no difficulty” category, placed in the “difficulty” category, or their status imputed based on their other characteristics. Any one of these four analytic strategies could introduce bias when IADL stages are applied in prevalence estimation. It is unclear how to best handle the resulting missing information since the true population prevalence of IADL limitation is unknown.

Our primary objective is to inform decisions about which strategies to use by estimating the presence, direction, and magnitude of differences in Medicare population prevalence estimation across each of these four assignment strategies. The secondary objective is to determine if “missing for other reasons” is disproportionally high among certain sub-populations in order to better recognize potential implications, magnitudes, and directions of biases when making inferences about the prevalence of IADL limitations in sub-populations. We hypothesize that there will be differences by age, gender, and perceived health status in comparing missing versus non-missing IADL.

## Methods

This study was approved by the Institutional Review Board at the University of Pennsylvania.

### Study population

This cross sectional study includes a nationally representative sample comprised of 13,879 civilian non-institutionalized persons ranging from 20 to 102 years of age at the time of their 2010 MCBS interview [10]. Details about these data are available elsewhere [11, 12]. Over 90 % of individuals (*n* = 12,433) reported for themselves. For the remaining 1446 individuals (8.9 %), proxy responses were included. Reasons for proxy use (% weighted do not add up to 100 % since sometimes multiple reasons were coded) were 16 (1.2 %) for being in hospital, 141 (13.5 %) for language problems, 629 (36.6 %) for not being able mentally, 428 (27.7 %) for not being capable physically, 489 (31.1 %) for having not kept medical records, 46 (2.9 %) for preferring proxy to answer, 176 (15.1 %) for being unavailable, and 24 (2.2 %) for other reasons.

### The IADLs

Sample persons (SPs) or their proxies were asked whether the SP had difficulty with performing any of six IADLs because of health or physical problems. The IADLs included using the telephone, managing money, doing light housework, preparing meals, shopping, and doing heavy housework [10]. Response options were: 1) no difficulty, 2) difficulty, 3) receives help, 4) does not perform. If a SP or proxy reported that the SP received help from another person to perform an activity then that person is considered to have difficulty. If the SP or proxy responded that the SP did not perform an activity, then the surveyor asked whether that was because of a health or physical problem or because of some other reason.

SPs were classified for each individual IADL into one of four response categories: (1) No limitation, (2) Difficulty, including the answer “does not do because of health or physical problems,” (3) Does not do for other than health or physical problems, or (4) totally missing information.

### Covariates

For the objective of studying missing information by subcategory, age categories were contrasted as less than 65 years of age and those 65 years of age and older.

Gender included male and female.

Perceived health status was reported by asking the SP or proxy whether the SP’s health was perceived as excellent, very good, good, fair, or poor compared to others the same age. Because of small cell sizes of the higher IADL stages, we dichotomized perceived health into better health and poorer health by combining excellent, very good, and good (better health), and by combining fair and poor (poorer health).

### Strategies for handling unrated IADLs

We compared four strategies of handling unrated IADLs from persons who reported that they do not do an IADL for other than physical or health reasons. The first strategy assigned the unrated IADLs to “difficulty” or “no difficulty” via multiple imputation. Multiple imputation relies on the assumption that the data are missing at random (MAR) which means the missingness is only related to observed, but not the missing information. Even if data are not MAR, if one can build a good predictive model for the missingness and one includes all important predictors of missingness in the multiple imputation logistic regression, then one can assume that the MAR assumption is reasonable [13]. In the multiple imputation strategy, we included age, gender, education, income, proxy use, marital status, general health perception, ADL status, IADL status, vision, hearing, communication, high cholesterol, social consequences of health, arthritis, amputation, dementia, Alzheimer’s disease, coronary heart disease, heart valve disorder, heart rhythm disturbance, congestive heart disease, hypertension, past myocardial infarction, other heart disease, broken hip in the past year, paralysis, stroke, mental retardation, Parkinson’s disease, diabetes, chronic obstructive pulmonary disease, arteriosclerosis, depression, other psychiatric disorders, skin cancer, and other cancers.

Given the wide array of information that predicts difficulty or no difficulty for IADL items, we believe the MAR assumption is reasonable. We used the SAS callable IVEware 0.2 (University of Michigan’s Survey Research Center, Ann Arbor) to perform multiple imputations. The software enabled multiple imputation of missing values by Sequential Regression Imputation Methods. The imputations were obtained by fitting a sequence of regression models and drawing values from the corresponding predictive distributions. Estimates were obtained by combining the results from the five multiply imputed datasets that were obtained. This is referred to as the “multiple imputation strategy.”

The second strategy assigned stages derived from the subsample remaining after excluding people who answered “did not do an IADL for other than health or physical reasons.” The presence or absence of systematic bias in this subsample relative to the entire population’s actual status is unknown. This option is referred to as the “complete case” strategy.

The third strategy assumed the SP would have no difficulties for the unrated IADLs. Because it is possible that some of those who report that non-performance is for a non-health reason may actually have physical or cognitive limitations that would render them unable to perform the task if attempted, this option might yield an underestimation of IADL difficulties in the population. Consequently, this stage assignment option is referred to as the “low prevalence” strategy.

The fourth strategy assumed the SP would have difficulty performing the unrated IADLs. Because it is possible that some of those who report that non-performance is for a non-health reason may actually not have physical or cognitive limitations that would render them unable to perform the task if attempted, this option might yield an overestimation of IADL difficulties in the population. Therefore, this stage assignment option is referred to as the “high prevalence” strategy.

### Statistical analyses

We compared the proportion of individuals who stated they do not do each IADL for other than health or physical problems by age, gender, and perceived health (better health versus poorer health).

We estimated the stage prevalence for each of the four strategies for the overall sample and by gender.

To further understand the implications of these four alternative assignment strategies to stage specification and their application to policy and health system questions, we looked at the association between each of the IADL stages assigned according to each strategy and perceived health. We calculated the proportions of people with poorer (fair combined with poor) perceived health in each of the IADL stages assigned according to the four strategies.

Since MCBS applied a complex survey design, we accounted for the design features such as unequal weights, clustering, and stratification in all analyses. Analyses were done in SAS Version 9.3 except for the multiple imputation.

## Results

Of the 13,879 total respondents, 1853 respondents (12.4 % weighted) stated they did not do one or more of the IADL tasks for reasons other than health or physical problems. In our data, 1347 did not do only one IADL, while 506 did not do more than one IADL. There were 41 respondents (0.3 % weighted) with totally missing data who did not rate one or more IADL for other reasons such as refusal to answer or answering “don’t know.” Thus, people with totally missing data did not respond to that particular survey question. Among them, 3 also did not do one or more IADL tasks for reasons other than health or physical problems.” The 41 respondents were only included in the multiple imputation analysis. Among the 13,879 included in our analytic sample, 54.8 % were female. Eighty-four percent of SPs were 65 years of age and older. There were 15.5 % who reported the SP’s health as excellent, 29.2 % as very good, 29.6 % as good, 17.5 % as fair, and 7.9 % as poor. There were 0.4 % of persons whose health was not rated.

With the exception of phone, there were gender differences for all of the IADLs, all showing men as more likely to say they do not do the task for non-health reasons (Table 1). Heavy housework was the task most often not done overall and phone use the least common left undone. For IADLs not done, there were smaller differences by age. There were significant differences by perceived health status for 5 of the 6 IADLs.

**Table 1:** The proportion of people who do not do individual Instrumental Activities of Daily Living (IADLs) for reasons other than health or physical problems reported by gender, age, and health status

	Phone *N* = 34	Money *N* = 408	Meal *N* = 553	Light housework *N* = 392	Shop *N* = 188	Heavy housework *N* = 1050
Gender						
Male (*N* = 6339)	17 (0.21 %)	244 (3.70 %)	439 (6.50 %)	341 (5.08 %)	142 (2.07 %)	614 (8.92 %)
Female (*N* = 7540)	17 (0.19 %)	164 (2.05 %)	114 (1.34 %)	51 (0.58 %)	46 (0.52 %)	436 (5.24 %)
*P*-values	0.6121	<.0001	<.0001	<.0001	<.0001	<.0001
Age						
<65 (*N* = 2436)	^a^	60 (2.54 %)	64 (2.46 %)	54 (2.25 %)	30 (1.23 %)	100 (3.70 %)
≥65 (*N* = 11443)	26 (0.18 %)	348 (2.84 %)	489 (3.90 %)	338 (2.68 %)	158 (1.22 %)	950 (7.51 %)
*P*-values	0.3589	0.1251	0.0002	0.0462	0.5629	<.0001
Perceived Health Status						
Excellent/Very Good/Good (*N* = 10219)	19 (0.14 %)	253 (2.31 %)	403 (3.57 %)	253 (2.22 %)	111 (0.93 %)	808 (7.11 %)
Fair/Poor (*N* = 3660)	15 (0.35 %)	155 (4.24 %)	150 (3.97 %)	139 (3.77 %)	77 (2.09 %)	242 (6.29 %)
*P*-values	0.0196	<.0001	0.7264	<.0001	<.0001	0.0107

^a^Cannot display percentage since cell size is less than 11

IADL stage prevalence estimates formulated by the low and high estimated population prevalence strategies tended to yield the lowest and highest population prevalence estimates of IADL difficulty, respectively (Table 2) overall and by gender. Stage prevalence estimates established from the complete case and imputation strategies were similar and generally fell between the two extremes, but closer to those estimated by the low prevalence (assume no difficulty) strategy.

**Table 2:** Population estimates by stages assigned according to alternative strategies for classifying those who do not do Instrumental Activities of Daily Living (IADLs) for reasons other than health or physical problems

IADL stage	Multiple imputation strategy	Complete case strategy^a^	Low prevalence estimate strategy^b^	High prevalence estimate strategy^c^
	% (std)	% (std)	% (std)	% (std)
Total Population				
0	57.2 (0.8)	57.9 (0.8)	60.0 (0.8)	52.2 (0.8)
I	19.6 (0.5)	18.1 (0.5)	17.6 (0.5)	20.2 (0.5)
II	9.8 (0.3)	9.6 (0.4)	9.2 (0.3)	11.9 (0.4)
III	11.1 (0.3)	11.9 (0.4)	10.8 (0.3)	13.0 (0.4)
IV	2.3 (0.2)	2.6 (0.2)	2.3 (0.1)	2.7 (0.2)
Gender = Male				
0	62.8 (0.9)	64.1 (0.9)	66.7 (0.8)	55.6 (0.9)
I	15.2 (0.6)	12.7 (0.6)	12.7 (0.5)	15.0 (0.6)
II	8.1 (0.4)	7.6 (0.3)	7.1 (0.3)	12.7 (0.4)
III	12.0 (0.5)	13.4 (0.6)	11.6 (0.5)	14.4 (0.6)
IV	1.9 (0.2)	2.2 (0.2)	1.9 (0.2)	2.4 (0.2)
Gender = Female				
0	52.6 (0.9)	53.1 (1.0)	54.6 (0.9)	49.4 (0.9)
I	23.4 (0.6)	22.2 (0.6)	21.7 (0.6)	24.6 (0.6)
II	11.0 (0.5)	11.1 (0.5)	10.9 (0.5)	11.3 (0.5)
III	10.4 (0.4)	10.7 (0.4)	10.2 (0.4)	11.8 (0.4)
IV	2.7 (0.2)	2.9 (0.2)	2.7 (0.2)	2.9 (0.2)

^a^Does not do for reason other than health or physical problem is not included in the subsample

^b^Does not do for reason other than health or physical problem coded as “no difficulty”

^c^Does not do for reason other than health or physical problem coded as “difficulty”

**Fig. 1:**
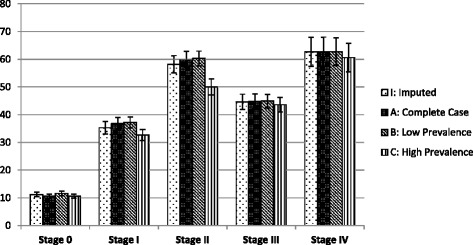
The proportion of individuals claiming fair or poor health (with standard error bars) by Instrumental Activity of Daily Living stages according to each strategy. Low prevalence estimation strategy: Does not do assigned to “no difficulty”. Complete case: Does not do excluded as missing. Multiple imputation: Does not do status predicted from other observed information. High prevalence estimation strategy: Does not do classified as “difficulty”

The expected association between the perception of fair or poor health and increasing stages of IADL disability was ordered and strong in all four stage assignment strategies, with stage III (the non-fitting stage) showing a drop (Fig. 1). At stage 0, there appeared to be little difference among the four strategies with regard to the proportion of individuals claiming fair or poor health. For stages I-IV, the proportions of people claiming fair or poor health were similar across the imputed, complete case, and low prevalence strategies. The greatest differences were in the high prevalence strategy seen at IADL-I and –II, where smaller proportions of people claimed fair or poor health than in the other three strategies.

## Discussion

Missing IADL information due to non-performance is a common problem in clinical practice, research, and policy around the world. We estimated IADL stage prevalence associated with four alternative strategies of handling information for IADL non-performers for reasons other than health or physical problems. Differences in prevalence estimation are not large when viewed at the population-level suggesting that IADL items can be used despite high rates of non-performance. Findings suggest that MCBS end-users could legitimately use complete case analysis in assigning stages when determining population prevalence estimates of IADL limitations. Overall, IADL stage prevalence estimates applying the complete case strategy of excluding the subsample reporting non-performance for non-health reasons are very close to the other estimation methods.

Results from the high prevalence staging assignment strategy are the most different from the other three methods. Comparing the high prevalence to the other three staging assignment strategies, we note that relatively smaller proportions of individuals within each stage claimed fair or poor health. It was only for this subsample claiming fair or poor health that the prevalence of disability estimated by the high prevalence strategy was slightly less than the other strategies. When the full range of self-rated health is considered, the high prevalence strategy yields higher rates as expected. This is because those for whom IADL(s) were seen as “not relevant” are classified as having “difficulty” when in reality they might not experience difficulty if they actually attempted to perform these activities. Conversely, there would be a small false negative misclassification difference within the low prevalence strategy. Stage prevalence estimates from the complete case subsample were most similar to the low prevalence and multiple imputation strategies. The implications of choosing one strategy over another appear to be minimal in population estimation because the prevalence estimates are so close among all assignment strategies. Consequently, when using MCBS data in most applications for policy end-users and others addressing population health and disability, it appears justified to exclude from analyses individuals for whom the performance of IADLs is not seen as relevant.

Assuming one could know the true prevalence for comparisons, the high prevalence strategy would be expected to slightly overestimate the proportion of people with limitations since it contains false positive assignments. In contrast, the low prevalence strategy would slightly underestimate the proportion of people with limitations in a population because it contains false negative assignments.

IADL items reflect abilities to cope with environmental demands [14]. The demands of these tasks are known to capture the functional consequences of early cognitive and physical impairments, and IADL function is usually lost before ADL function [15]. Thus, assessment of IADLs may identify incipient declines particularly in cognitive functioning in older adults who might otherwise appear capable and healthy [16]. Consequently, IADL is commonly used as a marker of functional decline in the elderly US population [17]. Yet, some individuals who did not do IADLs may have naturally sought others to take over these tasks at earlier stages of their disabling conditions. Thus, because of their life circumstances they did not need to address their difficulties until later and might be less inclined to acknowledge them in a survey.

Our findings that IADLs were more likely left unrated by men than by women is consistent with the finding that men who claim disabilities tend to have limitations that are more severe than women, although women are more likely than men to report some degree of disability [18, 19]. One possible explanation is that some men may be less willing than women to recognize mild mental and physical problems as a reason for not performing those tasks while at the same time, other men are truly reporting life-long roles that do not entail performance of those tasks. Based on the comparable performance of different classification approaches for men who report non-performance for non-health reasons, there does not appear to be a systematic bias that would raise concerns about using IADL measures of population health.

### Study limitations

This study has some limitations. Assumptions of Missing at Random on which multiple imputation is based cannot be tested. In addition, lack of a gold standard IADL measure makes it difficult to evaluate which method is further away from the truth. That said, our findings highlight very little differences in prevalence estimation across the four assignment strategies suggesting that any of the alternative strategies may be used in population prevalence estimation. We acknowledge that these findings are relevant primarily to the IADL questions as worded in surveys using wording similar to the MCBS. Although the IADL stages were derived from the MCBS data, it is likely that the results can be generalized to populations outside the US since performing IADLs is common for all people. Although the performance of IADLs may vary according to gender, social roles, and cultural traditions in different countries, reports of non-performance and missing data are expected to pose the same set of challenges for the study of non-U.S. populations. Finally, it is important for clinicians and researchers to understand person-specific issues inherent in doing functional assessment when addressing role functions. IADLs report average functioning in treatment populations.

## Conclusions

There have been longstanding concerns about the potential limitations of self-reported functional status versus the observed performance of activities. Yet, due to the high costs of measuring functional performance by observation, it is necessary to use self-reported survey information when assessing large populations. This work improves understanding of unrated survey responses as applied to population surveillance of IADLs. Findings highlight small differences in estimating IADL stage prevalence across 4 alternative ways of handling information from persons who state they do not do an IADL for reasons other than health. The resulting prevalence differences are trivial when applying MCBS to estimate IADL functioning of the Medicare population. This supports the reporting of IADL functioning despite relatively high rates of non-performance.

## Abbreviations

ADLs: activities of daily living
IADLs: instrumental activities of daily living
LSOA II: Second Longitudinal Study of Aging
MAR: missing at random
MCBS: Medicare Current Beneficiary Survey
SPs: sample persons
